# Prognostic value of interleukin-1 receptor antagonist gene polymorphism and cytomegalovirus seroprevalence in patients with coronary artery disease

**DOI:** 10.1186/1471-2261-5-10

**Published:** 2005-05-20

**Authors:** Dietrich Rothenbacher, Hermann Brenner, Thomas Mertens, Michael M Hoffmann, Albrecht Hoffmeister, Wolfgang Koenig

**Affiliations:** 1 Department of Epidemiology, The German Centre for Research on Ageing, University of Heidelberg, Germany; 2 Department of Virology, University of Ulm, Ulm, Germany; 3 Department of Clinical Chemistry, University of Freiburg, Freiburg, Germany; 4 Department of Internal Medicine II-Cardiology, University of Ulm Medical Center, Ulm, Germany

## Abstract

**Background:**

Chronic inflammatory stimuli such as cytomegalovirus (CMV) infection and various genetic polymorphisms determining the inflammatory response are assumed to be important risk factors in atherosclerosis. We investigated whether patients with stable coronary artery disease (CAD) and homozygous for allele 2 of the interleukin 1 receptor antagonist (IL-1RA) gene and seropositive for CMV represent a group particular susceptible for recurrent cardiovascular events.

**Methods:**

In a series of 300 consecutive patients with angiographically defined CAD a prospective follow-up was conducted (mean age 57.9 years, median follow-up time 38.2 months).

**Results:**

No statistically significant relationship was found between CMV serostatus and IL-1RN*2 (alone or in combination) and risk for future cardiovascular events (CVE). The hazard ratio (HR) for a CVE given positive CMV-serology and IL-1RN*2 was 1.07 (95% confidence interval (CI) 0.32–3.72) in the fully adjusted model compared to seronegative CMV patients not carrying the IL-1RN*2 allele. In this prospective cohort study involving 300 patients with angiographically defined CAD at baseline, homozygousity for allele 2 of the IL-1 RA and seropositivity to CMV alone and in combination were not associated with an increased risk for cardiovascular events during follow-up; in addition, combination of the CMV-seropositivity and IL-1RN*2 allele were not associated with a proinflammatory response

**Conclusion:**

Our study suggests that seropositivity to CMV and IL-1RA*2 genotype alone or in combination might not be a strong risk factor for recurrent cardiovascular events in patients with manifest CAD, and is not associated with levels of established inflammatory markers.

## Background

A chronic, low-grade inflammatory response plays a key role in atherosclerosis [1]. It has been suggested that chronic inflammatory stimuli originating from infectious agents may contribute to this low-grade inflammation and thus, might play a causal role in atherogenesis and disease progression in patients with manifest chronic coronary artery disease (CAD) [2]. The cytomegalovirus (CMV) infection has been discussed as potential culprit to cause atherosclerosis and especially to be involved in restenosis [3] as it, if once acquired, persists life long and may undergo periodic reactivation from latency [4].

Genes involved in the inflammatory response, such as the interleukin-1 receptor gene, which is polymorphic in nature, might be important in determining the response profile in patients with chronic inflammatory stimuli [5]. This gene is located on chromosome 2 in close prximity to genes coding for IL-1α and IL-1β. The main role of the IL-1 system is to mediate the early inflammatory reactions for protection against many different stimuli ranging from microbial colonization, infections, to malignant transformation. IL-1RA levels typically increases during the course of an inflammatory event so that an induced inflammation gets terminated. As persons homozygous for the allele 2 of the interleukin 1 receptor antagonist (IL-1RA) gene (Il-1RN*2) show a more prolonged and more severe immune response compared to persons with other allele constellations, subjects with the Il-1RN*2 allele and with evidence of chronic infection might be at particular high risk for cardiovascular events. However, data examining the combination of both factors are lacking.

We investigated whether homozygousity for the allele 2 of the IL-1RA and CMV-seropositivity might be associated with an increased risk for the development of cardiovascular events in patients with prevalent CAD to determine its prognostic value in secondary disease prevention in a prospective cohort study and whether this constellation was associated with various inflammatory response markers.

## Methods

### Study design and population

#### Baseline examination

Patients were recruited between October 1996 and November 1997 and were part of a case-control study investigating the role of infectious agents in primary CAD; details were reported elsewhere [6]. Briefly, the patient group consisted of 312 patients aged 40–68 years with clinically stable CAD who underwent elective coronary angiography in the Department of Cardiology at the University of Ulm Medical Centre during this period and who had a coronary stenosis of ≥ 50% of the luminal diameter of at least one major coronary artery. Patients with diagnosis of CAD older than 2 years, patients with acute coronary syndromes, and patients on anticoagulant therapy within the previous four weeks were excluded from the study.

At baseline, all study participants underwent a standardized interview carried out by specially trained interviewers. Participation was voluntary and written informed consent was obtained from each subject upon entry into the study. The study was approved by the ethics committee of the University of Ulm.

#### Follow-up examination

A follow-up (FU) of all patients with CAD was conducted between October 2000 and April 2001. A personal interview was conducted in the medical clinic (96.5%) or – if patients were not willing or able to follow the invitation – by phone by the same trained medical staff. Cardiovascular events (CVE) were defined by assessing the first occurrence of the following events during FU: cardiovascular death, nonfatal myocardial infarction, ischemic cerebrovascular event, and the need for coronary revascularization. All CVEs were validated by chart review.

### Laboratory methods

Venous blood was drawn under standardized conditions directly before diagnostic coronary angiography at the baseline examination. A complete blood cell count was done automatically by a Coulter STKS counter (Coulter, Krefeld, Germany). The remaining blood was centrifuged at 3,000 g for 10 minutes within 30 min after venipuncture, immediately aliquoted and frozen at -70°C until further analysis.

IgG antibodies against CMV were determined using a commercially available ELISA (CMV-IgG-ELISA PKS, medac, Wedel Germany) for the detection and quantitative determination of human IgG to cytomegalovirus according to the manufacturer's instructions (borderline zone 0.35–0.45 U/ml, only n = 3 (1%) of the samples had a borderline result).

IL-1RA gene polymorphisms were done by PCR as described in a similar study (7). Subjects homozygous for allele 2 of the IL-1RA gene (IL-1RN*2) were grouped against all other alleles constellations in the statistical analysis.

Additionally, the following markers of inflammation were determined by ELISA: Interleukin-6 (IL-6), and Tumor Necrosis Factor (TNF)-α (Quantikine, R&;D Systems, Wiesbaden, Germany), inter-cellular adhesion molecule (ICAM)-1 (Diaclone, Besancon, France). In addition, C- reactive protein (CRP) determinations were done by an immunoradiometric assay (range 0.05–10 mg/L) calibrated with the WHO reference standard 85/506. Serum amyloid A (SAA) was also determined by immunonephelometry (Dade Behring, Marburg, Germany). All laboratory analyses were done in a blinded fashion.

### Statistical analysis

Baseline demographic and clinical characteristics of cases were compared in a descriptive way. The association of anti-CMV IgG antibody titer (positive vs negative or borderline [1%]) and of the IL-1RA gene polymorphisms (IL-1RN*2 vs. others) with the occurrence of cardiovascular events during follow-up was analyzed by Chi-square (χ^2^) statistics. If expected cell frequencies were < 5, Fisher's Exact Test was used.

In addition the relation of CMV, of the IL-1RA gene polymorphisms and their combination with CVD events during follow-up was assessed by the Kaplan-Meier method and quantified by means of the log-rank test.

Multivariate Cox regression analysis was performed to determine the hazard ratio (HR) and 95% confidence intervals (CI) for future cardiovascular events taking potential confounding factors into account (controlling for age (years), gender, body mass index (BMI, kg/m^2^), school education, cigarette smoking, alcohol consumption, history of Myocardial infarction, history of hypertension, history of diabetes, statin intake, intake of aspirin, and intake of diuretics). The proportional hazard assumption were checked graphically. A general linear regression method was employed to calculate gender and age adjusted mean values (arithmetic, if skewed geometric) associated with CMV-seropositivity and the IL-1RN*2 allele compared to all other constellations. All analyses were carried out with the SAS statistical software package (SAS Institute, Version 8, Cary, North Carolina: SAS Institute, Inc).

## Results

In this prospective study a total of 300 (96.2%) out of 312 patients with a mean age of 57.9 years were followed for a median of 38.2 months (maximum 53.8 months). Twelve subjects could not be included in the follow-up as they refused to participate or they moved out of Germany and could not be contacted during follow-up. During the follow-up, 11 fatal and 49 non-fatal CVE occurred (20%) (5 patients died of a non-cardiac cause). Among CAD patients, who subsequently developed a non-fatal CVE, four patients suffered a myocardial infarction, seven an ischemic cerebrovascular event, and coronary revascularization was performed in 38 subjects.

Table one shows the main characteristics of the patients in patients with (n = 60) and without (n = 240) a CVE during follow-up. There were no statistically significant differences with respect to gender, age, mean body mass index, alcohol consumption habits and smoking status, and history of dyslipidemia, hypertension, diabetes and prior myocardial infarction. IN addition, coronary status and history of percutaneous coronary intervention (PCI) or coronary artery bypass graft (CABG) at baseline was also similar among the two groups.

**Table 1:** Baseline Characteristics of the Study Population (N = 300)

	**Patients With Cardiovascular Event (N = 60)**	**Patients Without Cardiovascular Event (N = 240)**	**p-value**
**Male**, n (%)	51 (85)	206 (85.8)	0.9
**Age **(y) mean ± SD	57.5 ± 7.7	58.0 ± 7.2	0.7
**Body mass index **(kg/m^2^) mean ± SD	27.0 ± 5.7	27.2 ± 3.5	0.6
**School education **<10 yrs, n (%)	46 (76.7)	165 (68.7)	0.8
**Daily alcohol consumption**, n (%)	18 (30)	71 (29.6)	0.9
**Smoking status:**			
Current, n (%)	1 (1.7)	25 (10.4)	
Past, n (%)	41 (68.3)	158 (65.8)	
Never, n (%)	18 (30)	57 (23.8)	0.07^a^
**History of:**			
Dyslipidemia, n (%)	37 (61.7)	167 (69.6)	0.2
Hypertension, n (%)	36 (60)	138 (57.5)	0.7
Diabetes, n (%)	9 (15)	32 (13.3)	0.7
**Prior myocardial infarction**, n (%)	40 (66.7)	144 (60.3)	0.4

^a ^= Fisher's Exact Test used

Table two shows the prevalence of the IL-1RN*2 allele, and the seroprevalence of CMV infection (results of the quantitative CMV analysis were not available in 5 patients). 8.3% and 10.4% (p = 0.6) of the patients with and without CVE respectively, were homozygous for the IL-1RN*2 allele (known to be associated with a more severe and prolonged inflammatory response).

**Table 2:** Prevalence of interleukin-1 receptor antagonist gene polymorphisms (IL-1RA) and serostatus of CMV and their combination in patients with and without cardiovascular events during follow-up

	**Patients with Cardiovascular Event (n = 60)**	**Patients without Cardiovascular Event (n = 240)**	**p-value**
**IL-1 RA**			
- other^a^	55 (91.7%)	215 (89.6%)	
- Il-1RN*2	5 (8.3%)	25 (10.4%)	0.6

**CMV-serostatusb**			
- negative	29 (49.2%)	110 (46.8%)	
- positive	30 (50.9%)	125 (53.2%)	0.7

**IL-1 RA and CMV serostatus**			
- IL-other^a ^and CMV-negative	27 (45.8%)	96 (40.9%)	
- IL-other^a ^and CMV-positive	27 (45.8%)	114 (48.5%)	
- Il-1RN*2 and CMV-negative	2 (3.4%)	14 (6.0%)	
- Il-1RN*2 and CMV-positive	3 (5.1%)	11 (4.7%)	0.8^c^

^a ^= all other alleles summarized (IL-1RN*4, IL-1RN*5, IL-1RN*3) except IL-RN*2

^b ^= in 6 patients quantitative determination of IgG was not available

^c ^= Fisher exact test

Overall, 52.7% of the patients were seropositive for CMV, and the distribution was similar in patients with and without CVE during follow-up (50.9% and 53.2% respectively, p = 0.7). There was also no difference in distribution among the two groups if IL-1RA genotype and CMV-seropositivity were combined: the IL-1RN*2 allele and CMV-seropositivity occurred in 5.1% of patients with CVE and in 4.7% of patients without, respectively (p = 0.8).

Table three shows the results of the multivariate analysis. Patients who had the IL-1RN*2 allele and patients seropositive for CMV (the latter independently also from titer values above the mean) showed no statistically significant increased risk for subsequent fatal or non-fatal CVE, in both, the partially as well as in the fully adjusted model.

**Table 3:** Distribution of factors and relation to cardiovascular events during follow-up and partly and fully adjusted hazard ratios for cardiovascular events associated with Interleukin-1 receptor antagonist gene polymorphisms (IL-1RA) and serostatus of CMV

**Factor**	**CVD-events during follow-up, **row % (^a^p-value)	**^b^Partly-adjusted hazard ratio **(95% confidence interval)	**^c^Fully-adjusted hazard ratio **(95% confidence interval)
**IL-1 RA**			
- other^d^	20.4%	1^reference^	1^reference^
- Il-1RN*2	16.3% (0.73)	1.1 (0.80–1.66)	1.03 (0.60–1.76)

**CMV-serostatus**			
- negative	20.7%	1^reference^	1^reference^
- positive		0.91 (0.55–1.52)	1.00 (0.59–1.72)
*- positive, titre < median*	*16.7%*	*0.74 (0.38–1.43)*	*0.78 (0.40–1.54)*
*- positive titre ≥ median*	*22.1% *(0.54)	*1.12 (0.61–2.05)*	*1.32 (0.69–2.52)*

**IL-1 RA and CMV serostatus**			
- IL-other^d ^and CMV-negative	21.9%	1^reference^	1^reference^
- IL-other^d ^and CMV-positive	19.1%	0.86 (0.50–1.45)	0.94 (0.54–1.64)
- Il-1RN*2 and CMV-negative	12.5%	0.56 (0.13–2.36)	0.37 (0.08 – 1.67)
- Il-1RN*2 and CMV-positive	21.4% (0.83)	0.96 (0.32–3.43)	0.95 (0.27 – 3.38)

^a ^= according to log-rank test

^b ^= adjusted for age and gender

^c ^= adjusted for age, gender, body mass index, school education, cigarette smoking, alcohol consumption, history of myocardial infarction, history of hypertension, history of diabetes, statin intake, intake of aspirin, intake of diuretics

^d ^= all other alleles

In addition, if both factors were combined no increased risk for CVE during follow-up was obtained (HR = 0.96 (95% CI 0.32–3.43) in the gender and age adjusted model, and HR = 0.095 (95% CI 0.27–3.38) in the fully adjusted model compared to CMV seronegative patients not having the IL-1RN*2 allele).

In addition, we found no statistically significant differences for mean values (geometric means with exception of ICAM-1 (arithmetic)) of CRP, SAA, Il-6, TNF-α, and ICAM-1 after adjustment for age and gender when CMV-seropositive patients with the IL-1RN*2 allele were compared to others (table 4).

**Table 4:** Mean concentrations^a,b ^of various markers of inflammation in patients with Il-1RN*2 and positive CMV-serostatus compared to the others

	**IL-1 RA and CMV serostatus**		
		
	- Il-1RN*2 and CMV-positive	**Others**	**p-value**
- CRP [mg/L] ^c^	0.92	1.58	0.5
- SAA [mg/L] ^c^	3.04	3.52	0.5
- Il-6 [pg/mL] ^c^	2.19	2.43	0.6
- TNF-α [pg/mL ] ^c^	2.37	2.53	0.6
- ICAM-1 [ng/mL]	456.6	537.1	0.08

^a ^= Adjusted for age and gender by general linear regression

^b ^= Arithmetic or if skewed ^c ^geometric means

## Discussion

In this prospective cohort study involving 300 patients with angiographically defined CAD at baseline, homozygousity for allele 2 of the IL-1 RA and seropositivity to CMV alone and in combination were not associated with an increased risk for cardiovascular events during follow-up; in addition, combination of the CMV-seropositivity and IL-1RN*2 allele were not associated with a proinflammatory response. Therefore, these data do not support the hypothesis that IL-1RN*2 genotype in combination with CMV seropositivity might be a strong risk factor for secondary cardiovascular events in patients with already prevalent cardiovascular disease.

These results are in contrast to several reports in the literature suggesting a positive association between CMV seropositivity and progression of atherosclerosis [8]. However, the earlier evidence was mostly based on small case-control studies with rather vague definitions of clinical endpoints and with little or no adjustment for potential confounders. Meanwhile, several large prospective seroepidemiological studies have shown no independent association for CMV and coronary heart disease [9,10]. But clearly more carefully conducted prospective studies in different patient populations are needed before definite conclusions can be drawn [11,12].

Evidence for a possible influence of the IL-1 RA polymorphism on CAD has been inconclusive so far [5]. One study reported a positive association with risk of CAD [13], another reported an positive association for the IL1RN*2 genotype with risk of restenosis in patients with CAD, if the population was restricted to a subgroup of patients with single vessel disease [7]. We found no association of CMV-serpositivity and IL1RN*2 allele with risk of CVE during follow-up, even if both factors were combined.

It has been described that subjects with the IL1RN*2 genotype might be more resistant against some infections [5]. Patients with the IL1RN*2 genotype indeed showed a lower CMV-seroprevalence than others in our population (55.2% vs. 46.6%), although this difference was not statistically significant.

Recently, it has been suggested that the detrimental effects of CMV-seroprevalence may be limited to subjects with an increased inflammatory response as characterized by high levels of CRP [14,15] or high IL-6 levels [16]. We did not find a positive association of CMV-seroprevalence with the occurrence of CVE confined to subjects with high levels (above the median) of IL-6 or CRP, although baseline IL-6 and CRP values (the latter only tentatively) showed a positive predictive association with the occurrence of cardiovascular events during follow-up. However, this association was not modified by CMV-serostatus (data not shown). As CMV-seroprevalence may be related to various adverse factors associated with CAD itself, issues of confounding have to be considered and may explain part of the discrepancies in the literature so far. There may be indeed a complicated interplay among established risk factors and exposure to infectious agents as recently suggested by our group [17].

When looking at the results of this study the following limitations should be considered: seroprevalence may not be a good marker for recurrent active infection with CMV, or even more relevant in this context, reactivation of CMV; cytomegalovirus-IgG does not diagnose active virus infection after primary infection. Furthermore, the current study cannot exclude a weak association between seroprevalence to CMV and IL-1RN*2 genotype and risk of CVE. However, the study had a power of 80 % to detect an RR of at least 1.6 and more associated with CMV-seropositivity alone, and an RR of 2.8 and more associated with combined CMV-seropositivity and presence of the IL-1RN*2 allele, respectively. In addition, lack of association of the CMV-seropositivity and IL-1RN*2 allele with established inflammatory marker levels, which play a key role in atherogenesis, are further reassuring the essentially negative findings of this study.

## Conclusion

Despite its limitations, our study suggests that seropositivity to CMV and IL-1RA*2 genotype alone or in combination might not be a strong risk factor for recurrent cardiovascular events in patients with manifest CAD, and is not associated with levels of established inflammatory markers.

## List of abbreviations

CABG = coronary artery bypass graft

CAD = coronary artery disease

CMV = Cytomegalovirus

CRP = C-reactive protein

CVE = cardiovascular events

FU = follow-up

ICAM-1 = intercellular adhesion molecule – 1

Il-6 = interleukin 6

IL-1RA = interleukin 1 receptor antagonist

OR = odds ratio

PCI = percutaneous coronary intervention

SAA = serum-amyloid- A

TNF-α = tumor-necrosis factors

-

## Competing interests

The author(s) declare that they have no competing interests.

## Authors' contributions

DR did the statistical analysis and wrote the initial draft. DR, HB, TM, AH, WK had the idea of the study and DR, HB, AH, WK did the study design and conduct. TM did the immunoassays. MMH carried out the molecular genetic part. All authors critically revised the MS and read and approved the final manuscript.

## Pre-publication history

The pre-publication history for this paper can be accessed here:


